# Intrinsic *tet*(L) sub-class in *Bacillus velezensis* and *Bacillus amyloliquefaciens* is associated with a reduced susceptibility toward tetracycline

**DOI:** 10.3389/fmicb.2022.966016

**Published:** 2022-08-04

**Authors:** Katrine Nøhr-Meldgaard, Carsten Struve, Hanne Ingmer, Yvonne Agersø

**Affiliations:** ^1^Chr. Hansen A/S, Hørsholm, Denmark; ^2^Department of Veterinary and Animal Sciences, University of Copenhagen, Copenhagen, Denmark

**Keywords:** antimicrobial, antibiotic, resistance evolution, *Bacillus*, intrinsic resistance, efflux pumps

## Abstract

Annotations of non-pathogenic bacterial genomes commonly reveal putative antibiotic resistance genes and the potential risks associated with such genes is challenging to assess. We have examined a putative tetracycline *tet*(L) gene (conferring low level tetracycline resistance), present in the majority of all publicly available genomes of the industrially important operational group *Bacillus amyloliquefaciens* including the species *B. amyloliquefaciens, Bacillus siamensis* and *Bacillus velezensis*. The aim was to examine the risk of transfer of the putative *tet*(L) in operational group *B. amyloliquefaciens* through phylogenetic and genomic position analysis. These analyses furthermore included *tet*(L) genes encoded by transferable plasmids and other Gram-positive and -negative bacteria, including *Bacillus subtilis*. Through phylogenetic analysis, we could group chromosomally and plasmid-encoded *tet*(L) genes into four phylogenetic clades. The chromosomally encoded putative *tet*(L) from operational group *B. amyloliquefaciens* formed a separate phylogenetic clade; was positioned in the same genomic region in the three species; was not flanked by mobile genetic elements and was not found in any other bacterial species suggesting that the gene has been present in a common ancestor before species differentiation and is intrinsic. Therefore the gene is not considered a safety concern, and the risk of transfer to and expression of resistance in other non-related species is considered negligible. We suggest a subgrouping of the *tet*(L) class into four groups (*tet*(L)1.1, *tet*(L)1.2 and *tet*(L)2.1, *tet*(L)2.2), corresponding with the phylogenetic grouping and *tet*(L) from operational group *B. amyloliquefaciens* referred to as *tet*(L)2.2. Phylogenetic analysis is a useful tool to correctly differentiate between intrinsic and acquired antibiotic resistance genes.

## Introduction

Antimicrobial resistance genes are widespread among bacteria and can transfer between bacterial species when associated with mobile genetic elements such as plasmids or transposons (Thomas and Nielsen, 2005). It is therefore a requirement by the European Food Safety Authority (EFSA) that bacteria intentionally introduced to food or feed are free of acquired antimicrobial resistance genes conferring resistance toward antimicrobial compounds that are considered highly or critically important for treatment of infections in humans by the World health Organization [EFSA panel on Additives and Products or Substances used in Animal Feed (FEEDAP), 2018; World Health Organisation (WHO), 2018]. European Food Safety Authority distinguishes between acquired and intrinsic resistance genes. Acquired resistance genes are considered a potential safety concern since they may spread between bacteria and increase the pool of resistance genes, which can compromise treatment of infections if acquired by pathogenic bacteria. Intrinsic resistance genes are generally conserved within a given bacterial species or subpopulation, are independent of antibiotic selection pressure, and spread clonally rather than horizontally (Cox and Wright, 2013). Therefore, the presence of intrinsic resistance genes is not considered a safety concern, and the risk of transfer to and expression of resistance in other non-related species is considered negligible [EFSA panel on Additives and Products or Substances used in Animal Feed (FEEDAP), 2018].

Species belonging to the *Bacillus* genus are often intentionally introduced to animal feed (Cutting, 2011). Genes with identity to antibiotic resistance genes have been found within the *Bacillus* genus (Agersø et al., 2018, 2019), however further analysis need to be performed in order to assess whether these genes are acquired or intrinsic.

The members of the genus *Bacillus* are common inhabitants of soil and aquatic sediment and are widely spread in every environment (Ruiz-García et al., 2005; Rooney et al., 2009). *Bacillus velezensis* and *Bacillus amyloliquefaciens* promote plant growth and display antifungal activities (Lopes et al., 2018). Both species have been granted the Qualified Presumption of Safety (QPS) status by EFSA (EFSA, 2020) and have been exploited industrially as microbial plant protectors (Lopes et al., 2018) and feed additives (Ngalimat et al., 2021). *B. amyloliquefaciens* and *B. velezensis* are closely related species and recently it was suggested, that the two species together with *B. amyloliquefaciens* subsp. *plantarum*, *Bacillus methylotrophicus* and *Bacillus siamensis* should form an “operational group *B. amyloliquefaciens*” based on the high identity between their *rpo*B genes (>98%), GC contents within a 0.5% range, tetranucleotide signatures, which is able to discriminate between species based on genomic fragments (Teeling et al., 2004) and average amino acid identity (AAI) values (Fan et al., 2017). However, the members of the group could not be classified into one single species based on the average nucleotide identity (ANI) and digital DNA–DNA hybridization (dDDH) calculation that was below the threshold proposed for species delineation (Fan et al., 2017). The taxon *B. velezensis* includes all the strains previously classified as *B. velezenesis*, *B. methylotrophicus* and *B. amyloliquefaciens* subsp. *plantarum* (Dunlap et al., 2016; Rabbee et al., 2019) whereas the species *B. amyloliquefaciens* and *B. siamensis* only include *B. amyloliquefaciens* and *B. siamensis*, respectively.

Previous work has shown that most *B. velezensis* and *B. amyloliquefaciens* strains harbor a gene that encodes a putative tetracycline efflux pump with highest identity to the *tet*(L) class of tetracycline resistance genes (Agersø et al., 2018). All *tet* efflux genes encode an ~46-kDa membrane-associated protein that export tetracycline from the cell and thereby reduces the intracellular concentration preventing the antimicrobial to reach its target, the ribosomes within the cell (Chopra and Roberts, 2001). The Tet efflux proteins are divided into six groups based on their amino acid identity and Tet(L) belong to group 2 together with Tet(K). Both Tet(L) and Tet(K) differ from the other Tet efflux classes by coding for proteins with 14 predicted transmembrane α-helices instead of 12, and are found primarily in Gram-positive bacteria positioned on small transmissible plasmids sometimes integrated into the chromosome (Gillespie et al., 1987; Sakaguchi et al., 1988).

The *tet*(L) class is highly diverse. It includes the plasmid-borne *tet*(L) genes from the *Staphylococcus aureus* pSTE1 plasmid and the *Bacillus stearothermophilus* pTHT15 plasmid which exhibit a high degree of sequence identity at nucleotide and amino acid level (99% and 98%, respectively) indicating a broad adaptation of these small plasmids (Chopra and Roberts, 2001). The *tet*(L) class furthermore include a chromosomal gene also classified as *tet*(L) from *B. subtilis* (AL009126) exhibit 81% amino acid sequence identity to the plasmid-encoded *tet*(L) genes (Schwarz et al., 1992), which is just at the limit of what would be considered as the same *tet* gene class (Chopra and Roberts, 2001). The putative *tet*(L)-like gene found in *B. amyloliquefaciens* and *B. velezensis* exhibit highest identity (87%) at nucleotide and amino acid level to the chromosomal *tet*(L) from *B. subtilis* (AL009126; Agersø et al., 2018). These chromosomally encoded efflux pumps are functional as they have been shown to cause reduced susceptibility toward tetracycline compared to strains without the gene or with a truncated gene (Sakaguchi and Shishido, 1988; Agersø et al., 2018).

The aim of this paper is to investigate the risk of mobilization of putative *tet*(L) genes in *B. velezensis* and *B. amyloliquefaciens*. This was addressed by phylogenetic and *in silico* genome analysis of the genetic regions flanking the two genes as well as by their similarity to *tet*(L) genes encoded by other Gram-positive and Gram-negative bacteria. We also discuss the current tetracycline resistance gene classification system (Chopra and Roberts, 2001), which do not consider the phylogenetic relationship of genes.

## Materials and methods

### Bacterial genomes

The genomes analyzed in the study include all publicly available whole-genome sequences from *B. velezensis*, *B. amyloliquefaciens*, and *B. siamensis*, including the three type strains KCTC 13012, DSM7 and KCTC 13613 respectively, which were available in the NCBI microbial genome database on 13 January 2021. Also, previously sequenced *B. velezensis* and *B. amyloliquefaciens* strains from the culture collection at Chr. Hansen A/S was included in the study (Agersø et al., 2018). In total 72 *B. velezensis*, 13 *B. amyloliquefaciens*, and 7 *B. siamensis* genomes were included in the study (Supplementary Table S1). Nine of the genomes originates from strains from the Chr. Hansen’s culture collection for which minimal inhibitory concentration values have previously been determined (Agersø et al., 2018).

The *B. amyloliquefaciens, B. velezensis,* and *B. siamensis* strains included in the present study (Supplementary Table S1) are expected to be a good representation of the diversity of the species, as they have been isolated from different geographical areas, time points and sources that covers the different habitats of the species.

### Genome quality

The sequence quality of the genomes was assessed by checking the number of contigs, overall coverage, GC content and genome size. The genomes included in the study consisted of either contigs or complete genomes and exhibited a contig number below 70 and an average coverage above ≥40×, which was considered acceptable for further genome analysis.

### Multi locus sequence typing and species identification through genome-based taxonomy

Multi locus sequence typing was performed using the PubMLST typing database for *Bacillus subtilis*.^1^ When the species corresponding with the sequence type did not match with the species at NCBI, the Type (Strain) Genome Server (TYGS),^2^ which rely on core genome phylogeny, DNA:DNA hybridization values and differences in GC% content, was used to confirm the species identified by PubMLST.

This approach reclassified 24 *B. amyloliquefaciens* strains, as identified by NCBI, to *B. velezensis*. *B. amyloliquefaciens* subsp. *plantarum* now belong to the *B. velezensis* taxon, which could explain the reclassification of several *B. amyloliquefaciens* strains (Dunlap et al., 2016).

The size and GC content of the *B. velezensis, B. amyloliquefaciens,* and *B. siamensis* genomes are in the range of what previously have been shown for these species (Supplementary Table S1; Fan et al., 2017).

### Screening for *tet*(L)

At least one of each *tet*(L) gene homologue was included in the study. If one homologue was found in several species, one from each species was included. The following papers with reference to *tet*(L) in bacterial species were found searching PubMed (Takayuki et al., 1985; Lacks et al., 1986; Sakaguchi et al., 1988; Palva et al., 1990; Schwarz et al., 1992; Amano et al., 1993; Platteeuw et al., 1995; Kadlec and Schwarz, 2009; Phelan et al., 2011; Tang et al., 2020). To ensure all known *tet*(L) genes and species with *tet*(L) were included, BLAST searches of *tet*(L) genes (FN377602, M29725, X51366, HM235948, U17153, AL009126, D0006, X60828, M11036) were performed against the NCBI NR database. The *tet*(L) genes and proteins as well as genomes (if available) were extracted either from GenBank files downloaded from the NCBI microbial genome database or from RAST annotated genomes. The Rapid Annotation using Subsystems Technology (RAST) tk server was used with default settings to annotate genomes (Aziz et al., 2008; Overbeek et al., 2014). GenBank and RAST annotated files were imported in to CLC Genomics Workbench 20 (Qiagen Bioinformatics, Aarhus, Denmark).

### Examination of sequences flanking *tet*(L)

The flanking regions of *tet*(L) in the different species were examined in order to determine whether the gene was present on, e.g., a plasmid, a transposon or not associated with mobile genetic elements. In order to examine the *tet*(L) genomic position, the genome alignment visualization tool MAUVE was used to align RAST annotated genomes (Darling et al., 2004).

All the downloaded genomes were annotated using the Rapid Annotation using Subsystems Technology (RAST) tk server with default settings (Aziz et al., 2008; Overbeek et al., 2014) and imported to CLC Genomics Workbench 20 (Qiagen Bioinformatics, Aarhus, Denmark).

The ResFinder database (Zankari et al., 2012) was used to search for the presence of *tet*(L) in the annotated genomes and the annotated genes flanking the *tet*(L) genes were examined. The ResFinder database was downloaded and imported into CLC Genomics Workbench 20.0 on the 29 September, 2020. The assembled contigs of each strain were joined using the join function in CLC and the joined contigs were screened for resistance genes against the ResFinder database, with a minimum word size of 11 and a maximum *E*-value of 1.0E-10. GC content of *tet*(L) and other genes was assessed by employing the DNA/RNA GC Content Calculator at ENDMEMO (ENDMEMO, 2020).

### *tet*(L) nucleotide and amino acid phylogenetic analysis

*tet*(L) nucleotide and protein sequences were extracted from the annotated genomes. ClustalX2 (Larkin et al., 2007) was used to perform a pairwise multiple alignment of *tet*(L) sequences (Higgins and Sharp, 1988) and BioEdit (Hall, 1999) was used to remove gaps and unpaired ends. The nucleotide phylogeny was built by evolutionary analysis by the Maximum Likelihood method and Tamura-Nei model by MEGA X (Tamura and Nei, 1993; Kumar et al., 2018) and the amino acid phylogeny was built by evolutionary analysis by Maximum Likelihood method and JTT matrix-based model also by MEGA X (Jones et al., 1992; Kumar et al., 2018).

### Core genome phylogeny of *Bacillus amyloliquefaciens*, *Bacillus velezensis*, and *Bacillus siamensis*

The genomes were annotated by Prokka, which annotates genomes through the use of different tools including Prodigal (coding sequences), RNAmmer (Ribosomal RNA genes), Aragorn (Transfer RNA genes), SignalP (Signal leader peptides) and Infernal (Non-coding RNA; Seemann, 2014). Prokka annotation is a requirement for using Roary, since the.gff file (file containing sequences and annotations) provided by Prokka is used by Roary to create a multi-FASTA alignment of all the core genes (Page et al., 2015). Roary was set to perform nucleotide alignment using MAFFT and a Blastp percentage identity at 80% (Katoh, 2002). FastTree was used to produce an approximately-maximum-likelihood phylogenetic tree from the core gene alignment file, which was visualized by MEGA X (Price et al., 2009, 2010; Kumar et al., 2018) and edited in FigTree v1.4.4.^3^

### Transmembrane domain prediction

Prediction of transmembrane domains was performed using the Constrained Consensus TOPology (CCTOP) prediction server.^4^

## Results and discussion

### *tet*(L) occurrence in Gram-positive and Gram-negative bacteria

The presence of *tet*(L) in bacterial species were searched for in previously published papers using PubMed and BLASTn searches against GenBank accession numbers (FN377602, M29725, X51366, HM235948, U17153, AL009126, D0006, X60828, M11036).

*tet*(L) was mainly found on plasmids but also chromosomally in Gram-positive bacterial species including *B. subtilis, B. amyloliquefaciens, B. siamensis* and *B. velezensis* (Supplementary Table S2), which is in accordance with previous knowledge of group 2 efflux pumps that include *tet*(L) and *tet*(K) (Chopra and Roberts, 2001; Agersø et al., 2018). Furthermore *tet*(L) was found on the chromosome of several strains of the Gram-negative species *Campylobacter jejuni* where it was associated with mobile genetic elements and a *C. jejuni* plasmid (Tang et al., 2020; Supplementary Table S2), indicating that *tet*(L) has a wide host range.

A 1377 bp putative *tet*(L) gene was found on the chromosome of the majority of the publicly available genomes of *B. amyloliquefaciens* [80% (8/10)]*, B. velezensis* [90.27% (65/72)] *and B. siamensis* [100% (7/7); Supplementary Table S2]. This is in accordance with previous published work (Agersø et al., 2018) that showed the presence of the putative *tet*(L)-like gene in *B. velezensis* and *B. amyloliquefaciens* correlated with reduced tetracycline susceptibility.

The majority of the *tet*(L) genes (Supplementary Table S2) exhibited the alternative GTG start codon, including the ones encoded by *B. amyloliquefaciens, B. velezensis* and *B. siamensis*. A few exhibited the ATG start codon (FN377602, KP036966, KY400493, EF605268) and most of these (FN377602, KP036966, KY400493) also differed in length (1,203–1,383 bp) compared to the *tet*(L) genes with GTG start codon. GTG start codons have been shown to be used by 9% of the coding sequences in *B. subtilis* (Rocha et al., 1999) and be associated with a less efficient translation compared with ATG (Vellanoweth and Rabinowitz, 1992).

The number of transmembrane domains were examined, and the results showed that all the Tet(L)-like proteins encoded by *B. amyloliquefaciens, B. velezensis*, and *B. siamensis* exhibited 14 predicted transmembrane domains, which previously have been shown for Tet(L) and Tet(K) (Chopra and Roberts, 2001). The majority of the Tet(L) proteins encoded by the other Gram-positive and Gram-negative bacteria were also predicted to have 14 transmembrane domains, except the smaller Tet(L) proteins with ATG start codon found in *Enterococccus faecium* KN9 (KP036966) and *Streptococcus suis* 74911-8 (KY400493), which were predicted to have 13 and 12 transmembrane domains, respectively (Supplementary Table S1). This suggest that Tet(L) and Tet(L)-like proteins from *B. amyloliquefaciens, B. velezensis* and *B. siamensis* share a similar structure.

### *Bacillus amyloliquefaciens*, *Bacillus velezensis*, and *Bacillus siamensis tet*(L) differ from *tet*(L) in other Gram-positive and Gram-negative bacteria

**Figure 1:**
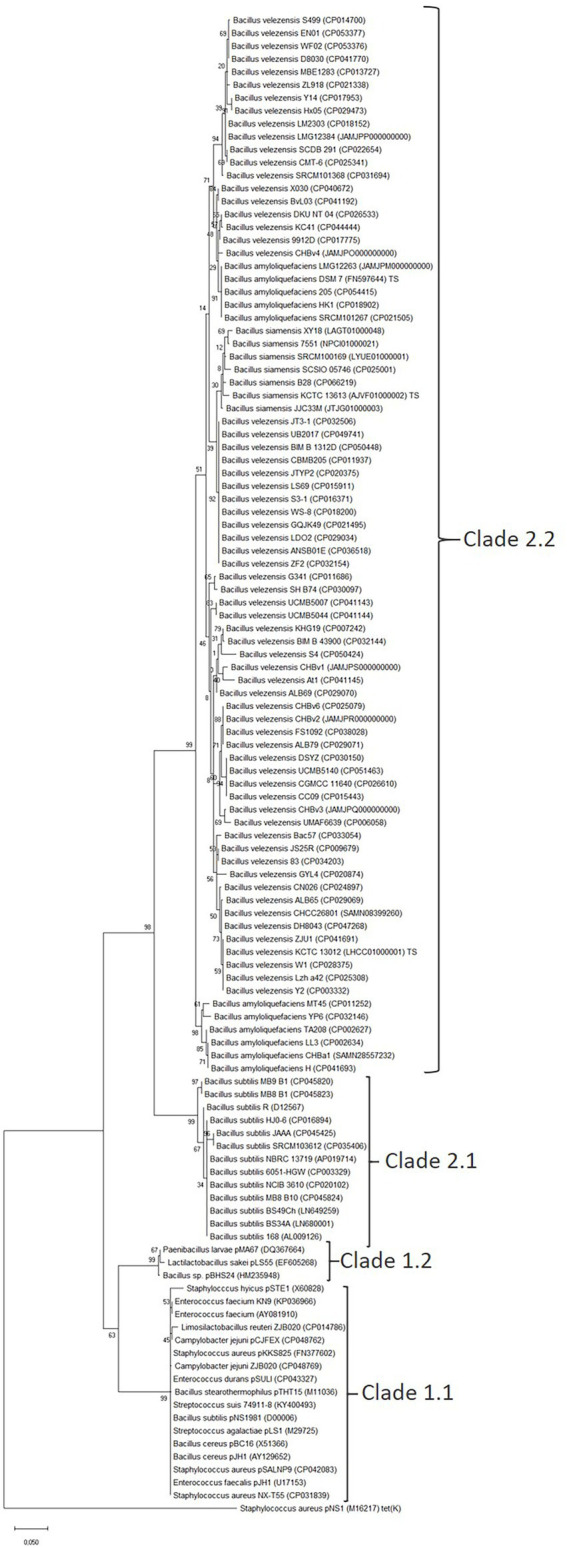
Tet(L) protein phylogenetic tree. The tree was built by Evolutionary analysis by Maximum Likelihood method and JTT matrix model (Jones et al., 1992; Kumar et al., 2018). The branch lengths are measured in the number of substitutions per site. Strains and GenBank accession number are given for each Tet(L) protein. The phylogenetic tree was rooted with the *Staphylococcus aureus* Tet(K) protein (M16217) as an outgroup. The numbers at the nodes are bootstrap values (1–100).

The phylogenetic relationship between all available *tet*(L) genes (Supplementary Table S2) was examined at both the nucleotide (Supplementary Figure S1) and protein level (Figure 1).

The phylogenetic analysis separates the Tet(L) proteins into four clades (Table 1). The four clades are well supported by high bootstrap values (99–63). The lowest (63) supports the splitting of clade 1.1 and 1.2. However, the Tet(L) proteins in clade 1.2 exhibit around 87%–88% identity to the plasmid-encoded Tet(L) from clade 1.1, suggesting that the subgrouping is valid.

**Table 1:** Nucleotide and amino acid identity of the four phylogenetic Tet(L) clades.

**Clade number**	**Clade feature**	**Species**	**Identity (%) to plasmid-encoded *tet*(L) (CP042083)**	**Identity (%) to *B. subtilis tet*(L) (AL009126)**
1.1	Plasmid borne	*Bacillus cereus, Bacillus stearothermophilus, Bacillus subtilis, Enterococcus faecalis, Enterococcus faecium, Staphylococcus aureus, Staphylococcus hyicus, Streptococcus agalactiae, Campylobacter jejuni*	99.06%–100% (98.25%–100%)^a^	79.88%–80.53% (81.40%–81.62%)
	Chromosomal encoded	*Streptococcus suis, Lactobacillus reuteri, C. jejuni, S. aureus*	99.92%–100% (98.69%–100%)	79.88%–80.53% (80.45%–81.62%)
1.2	Plasmid encoded	*Bacillus* sp., *Lactilactobacillus sakei, Paenibacillus larvae*	89.45%–89.68% (87.77%–88.21%)	83.06%–83.31% (82.42%–82.68%)
2.1	Chromosome *B. subtilis*	*B. subtilis*	80.32%–80.53% (81.40%–81.62%)	98.40%–100% (98.47%–100%)
2.2	Chromosome *Bacillus amyloliquefaciens, Bacillus velezensis, B. siamensis*	*B. amyloliquefaciens, B. velezensis, Bacillus siamensis*	78.57%–79.69% (79.43%–81.40%)	86.50%–87.97% (86.21%–88.18%)

a Percentage in parenthesis is amino acid identity.

Clade 1.1 includes both plasmid and chromosomally encoded Tet(L) proteins from both Gram-positive and Gram-negative bacteria associated with mobile genetic elements. Clade 1.1 Tet(L) proteins will from here be referred to Tet(L)1.1.

Most of the Tet(L)1.1 proteins found in clade 1.1 are located on plasmids and originate from several different bacterial genera and species (Table 1). The plasmids can be separated into three groups based on size and genetic context of the plasmid.

The plasmids from *B. stearothermophilus* (M11036)*, B. subtilis* (D00006)*, Bacillus cereus* (X51366) and *Staphylococcus hyicus* (X60828) were all small (1616–1,644 bp), showed a high degree of homology at nucleotide level (98.64%–99.94% identity, 98%–100% coverage) and encoded the *tet*(L)1.1 gene together with identical tetracycline resistance efflux system leader peptide, which is involved with inducible expression of tetracycline resistance in *B. subtilis* (Sakaguchi et al., 1988). *tet*(L) are often found on these small transmissible plasmids that have the ability to integrate into the chromosome of staphylococci and *B. subtilis* and even larger staphylococcal plasmids (Chopra and Roberts, 2001).

The plasmid from *Streptococcus agalactiae* (M29725) and *B. cereus* (AY129652), were of medium size (4,408 and 3,068 bp, respectively), only contained *tet*(L)1.1, the tetracycline resistance efflux system leader peptide sequence and genes involved in replication and plasmid transfer.

**Figure 2:**
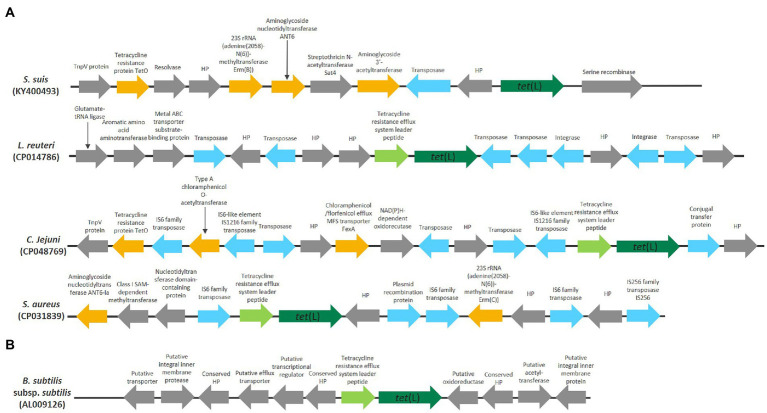
A schematic representation of the genomic position of chromosomally encoded *tet*(L) in Gram-positive and Gram-negative bacteria. HP is used as abbreviation for hypothetical proteins. Genes encoding antibiotic resistance genes, besides *tet*(L), are colored orange, genes related to mobility are colored blue and tetracycline resistance efflux system leader peptide are colored light green. **(A)** Genomic position of clade 1.1 *tet*(L). **(B)** Genomic position of clade 2.1 *tet*(L).

The plasmids from two *S. aureus* strains (CP042083, FN377602), *Enterococcus durans* (CP043327) and *C. jejuni* (CP048762) all encoded several different antibiotic resistance genes and were generally large (9,395, 14,362, 60,228, and 48,003 bp, respectively). Both the plasmid from *S. aureus* (CP042083) and *C. jejuni* (CP048762) encoded antibiotic resistance genes that were also observed in the near proximity of the clade 1.1 chromosomally encoded *tet*(L)1.1 in *S. aureus* NX-T55 (CP031839) and *C. jejuni* ZJB020 (CP048769; Figure 2A). This indicates that parts of these bigger *tet*(L)1.1-carrying plasmids might have the ability to integrate into the chromosome, which previously have been reported for *tet*(L) and *tet*(K) genes (Gillespie et al., 1987; Sakaguchi et al., 1988).

One *tet*(L)1.1 gene was encoded on a transposon in *Enterococcus faecium* (KP036966), together with a ribosomal protection gene *tet*(M) with 99.79% nucleotide identity to the plasmid-encoded *tet*(M) from *Neisseria meningitidis* (GenBank accession number X75073) and a streptomycin adenylase with 100% identity at nucleotide level to the plasmid-encoded streptomycin adenylase in *Lactococcus lactis* sp. *lactis* (X92946). The transposon also encoded transposases, integrase genes and a tetracycline resistance efflux system leader peptide.

The chromosomally encoded *tet*(L)1.1 genes in *S. suis* (KY400493)*, Lactobacillus reuteri* (CP014786)*, C. jejuni* (CP048769) and *S. aureus* (CP031839) are positioned in a genomic region with several genes involved with mobility and genes coding for antibiotic resistance genes (Figure 2A). Both *S. suis* (KY400493) and *C. jejuni* (CP048769) encoded a *tet*(O) gene with high nucleotide identity (98.70% identity with 100% coverage and 94.76% identity with 96% coverage, respectively) to *tet*(O) (GenBank accession number M18896). *tet*(L)1.1 was preceded by a tetracycline resistance efflux system leader peptide sequence in CP014786, CP048769, CP031839.

The presence of both mobile genetic elements and antibiotic resistance genes in the near vicinity of the clade 1.1 chromosomally encoded *tet*(L)1.1 genes indicates that in these strains this genomic region exhibits a high degree of plasticity, which is in accordance with the similarity to the clade 1.1 plasmid-encoded Tet(L)1.1 proteins. Together, this indicate that *tet*(L)1.1 has been acquired horizontally by these strains.

The fact that both plasmid and chromosomally encoded Tet(L)1.1 genes associated with mobile genetic elements cluster together in clade 1.1 is supported by the findings that *tet*(L) and *tet*(K) genes from plasmids have been found integrated into the chromosome of staphylococci (Gillespie et al., 1987) and *B. subtilis* (Sakaguchi et al., 1988).

Clade 1.2 includes the plasmid-encoded Tet(L) from *Bacillus* sp. (HM235948), *Lactilactobacillus sakei* Rits9 (EF605268) and *Paenibacillus larvae* (DQ367664). Clade 1.2 Tet(L) proteins will from here be referred to Tet(L)1.2.

The three plasmids were of medium size (5030–5,031 bp) and they contained *tet*(L) together with genes involved in replication, plasmid transfer and encoded identical tetracycline resistance efflux system leader peptides. The plasmids resembled the clade 1.1 medium sized plasmids from *S. agalactiae* (M29725) and *B. cereus* (AY129652) with regards to size and genetic context, but differed by exhibiting *tet*(L) with a reduced nucleotide and amino acid identity compared to clade 1.1 *tet*(L)0.1.1 (Table 1).

Clade 2.1 include the chromosomal Tet(L) proteins found in *B. subtilis,* which cluster separate from Tet(L)1.1 and Tet(L)1.2 proteins found on plasmids or chromosomally associated with mobile genetic elements, including the Tet(L)1.2 plasmid-encoded protein from *Bacillus* sp. Clade 2.1 Tet(L) exhibit an amino acid identity to the Tet(L)1.1, which is just above the 80% limit that define if tetracycline resistance genes belong to the same class (Table 1; Chopra and Roberts, 2001). Clade 2.1 Tet(L) proteins will from here be referred to as Tet(L)2.1.

The phylogenetic analysis furthermore showed that the *B. subtilis* chromosomally encoded Tet(L)2.1 were closely related to the Tet(L)-like proteins from *B. amyloliquefaciens, B. velezensis* and *B. siamensis* than the plasmid-encoded Tet(L)1.1 and Tet(L)1.2.

The *B. subtilis tet*(L)2.1 gene was found in about 60% of the publicly available *B. subtilis* genomes and no mobile genetic elements were found in the near vicinity of *tet*(L)2.1 in *B. subtilis* subsp. *subtilis* strain 168 (AL009126; Figure 2B). The *tet*(L)2.1 in the studied *B. subtilis* strains are positioned within a conserved genomic region, suggesting that *tet*(L) in *B. subtilis* is conserved within a subpopulation, and has not been recently acquired.

Clade 2.2 consists of the putative Tet(L) proteins from operational group *B. amyloliquefaciens including B. amyloliquefaciens, B. velezensis* and *B. siamensis*, which formed a separate clade from the Tet(L)1.1, Tet(L)1.2, and Tet(L)2.1 from other Gram-positive and Gram-negative bacteria (Figure 1). Clade 2.2 Tet(L) also exhibit a nucleotide and amino acid identity around 80% (Table 1). Clade 2.2 Tet(L) proteins will be referred to Tet(L)2.2 from now on.

**Figure 3:**
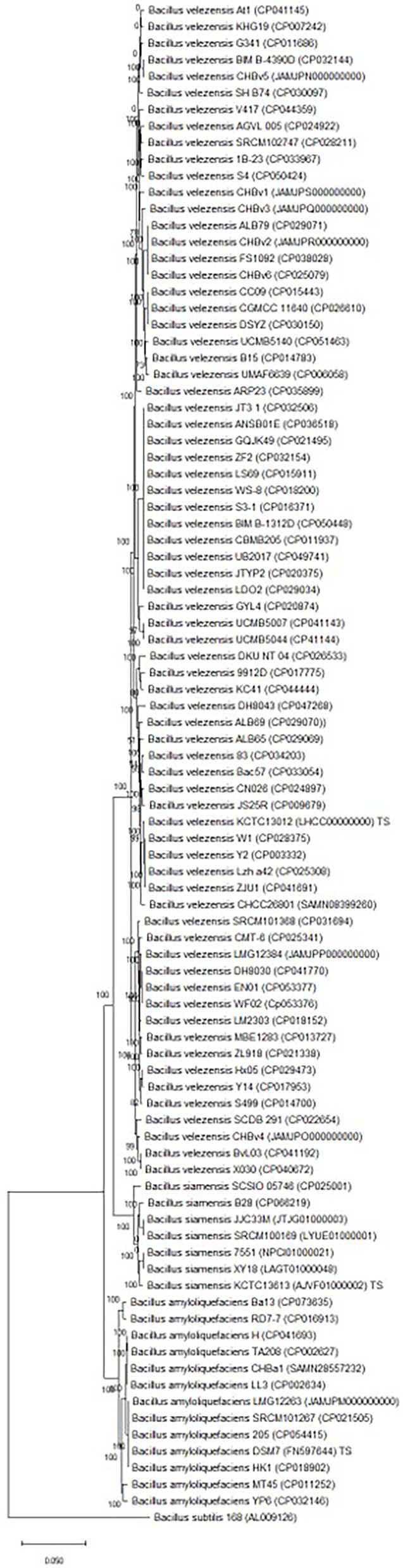
Core genome phylogenetic tree based on 642 core genes and include *B. veleznesis, Bacillus amyloliquefaciens* and *B. siamensis*. The tree is rooted with the *Bacillus subtilis* type strain 168 (GenBank accession number AL009126). The numbers at the nodes are bootstrap values (1–100).

Tet(L)2.2 generally follows the phylogeny of the operational group *B. amyloliquefaciens* (Figure 3; Fan et al., 2017), suggesting that the *tet*(L)2.2 has been present in a common ancestor of operational group *B. amyloliquefaciens* before species differentiation. The Tet(L)2.2 proteins from the *B. amyloliquefaciens* genomes were split in two groups. The first group includes the type strain Tet(L)2.2 (DSM7) together with Tet(L)2.2 from LMG12263 and these exhibit a higher nucleotide identity (97.39%–97.46% identity with 100% coverage) to the *tet*(L)2.2 gene encoded by the *B. velezensis* type strain KCTC13012 compared to the other group of *B. amyloliquefaciens* strains (MT45, YP6, H, CHBa1, TA208, LL3; 94.48%–94.92% identity with 100% coverage). Furthermore, the *B. siamensis* Tet(L)2.2 proteins are also phylogenetically positioned within the *B. velezensis* Tet(L)2.2 proteins (Figure 1), even though the strains have been correctly identified at species level by employing core genome analysis (Figure 3). This could suggest that a recombination event have taken place between the *B. amyloliquefaciens* type strain group, *B. siamensis* and *B. velezensis.*

The phylogenetic analysis overall shows that the *B. velezensis*, *B. amyloliquefaciens* and *B. siamensis* Tet(L)2.2 proteins are more closely related to the chromosomally encoded Tet(L)2.1 from a subpopulation of *B. subtilis* than *tet*(L)1.1 and *tet*(L)1.2 from other Gram-positive and Gram-negative bacteria associated with mobile genetic elements (Figure 1).

Besides the phylogenetic splitting of Tet(L)2.2 from Tet(L)1.1, Tet(L)1.2 and Tet(L)2.1, the *tet*(L)2.2 genes from *B. amyloqiuefaciens*, *B. velezensis* and *B. siamensis* exhibit a GC content of 43.1%–44.0% (Supplementary Table S2), which is slightly lower than the overall genome GC content (*B. amyloliquefaciens*: 45.6%–46.3%, *B. velezensis*: 45.2%–46.7%, *B. siamensis* 45.7%–46.4%; Supplementary Table S1). GC content of genes tends to be higher in highly expressed genes (Wuitschick and Karrer, 1999) and it could be speculated that the slightly lower GC content of *tet*(L)2.2 compared to the genome is due to downregulation of the gene. Furthermore, the GC content of the *tet*(L)2.2 is higher than the GC content of the *tet*(L)1.1 and *tet*(L)1.2 genes (35.1%–35.9%; Supplementary Table S2) and the *tet*(L)2.1 genes in *B. subtilis* (39.6%–40.2%).

A previous study have shown that *tet*(L) encoding *B. amyloliquefaciens* and *B. velezensis* exhibit tetracycline MIC range of 2–16 mg/L (Agersø et al., 2018), which is comparable with the tetracycline MIC of 8 mg/L for *B. subtilis* in clade 2.1 (Amano et al., 1991), but lower than clade 1.1 and 1.2 plasmid-borne *tet*(L) that have been reported to be 75 mg/L and 64 mg/L (Kadlec and Schwarz, 2009; Phelan et al., 2011).

Together, this indicates the *tet*(L)2.2 gene encoded by *B. amyloliquefaciens, B. velezensis,* and *B. siamensis* differ from *tet*(L)1.1, *tet*(L)1.2 and *tet*(L)2.1 encoded by other Gram-positive and Gram-negative bacteria, further supporting a divergent evolution of the *tet*(L) antibiotic resistance gene into subclasses.

### Truncated versions of the *tet*(L)2.2 gene in *Bacillus amyloliquefaciens* and *Bacillus velezensis*

The majority of the examined *B. amyloliquefaciens* (8/10) and *B. velezensis* (65/72) genomes encodes a full length version of the *tet*(L) gene. However, one *B. amyloliquefaciens* strain (Ba13) and two *B. veleznesis* strains (AGVL-005 and V417) does not encode a *tet*(L)2.2 gene (Supplementary Table S2). Furthermore, one *B. amyloliquefaciens* strain (RD7-7) and six *B. velezensis* strains (1B-23, SRCM102747, S141, CHBv5, MBE1283, B15) encodes a truncated version of the *tet*(L)2.2 gene, where the truncated *B. amyloliquefaciens tet*(L)2.2 gene aligns to the first part of *tet*(L)2.2 and the truncated *B. velezensis tet*(L)2.2 genes all aligns to the last part. Only one strain, namely *B. velezensis* ARP23, exhibits a truncated *tet*(L)2.2 gene as a results of a stop codon after 1,032 bp. The absence of *tet*(L) and presence of truncated versions have previously been reported in *B. amyloliquefaciens* and *B. velezensis* (Agersø et al., 2018).

The strains with a truncated *tet*(L)2.2 gene or no *tet*(L)2.2 gene have all been isolated from plant material or plant rhizosphere between the year of 2009 and 2017 but originate from different countries, belong to different sequence types (Supplementary Table S1) and are positioned in different clades in the core genome phylogenetic tree (Figure 3). It could be speculated that absence or truncation of the putative *tet*(L)2.2 gene might confer a specific function to strains related to plants.

### Position of the *tet*(L)2.2 gene in *Bacillus amyloliquefaciens*, *Bacillus velezensis*, and *Bacillus siamensis*

**Figure 4:**
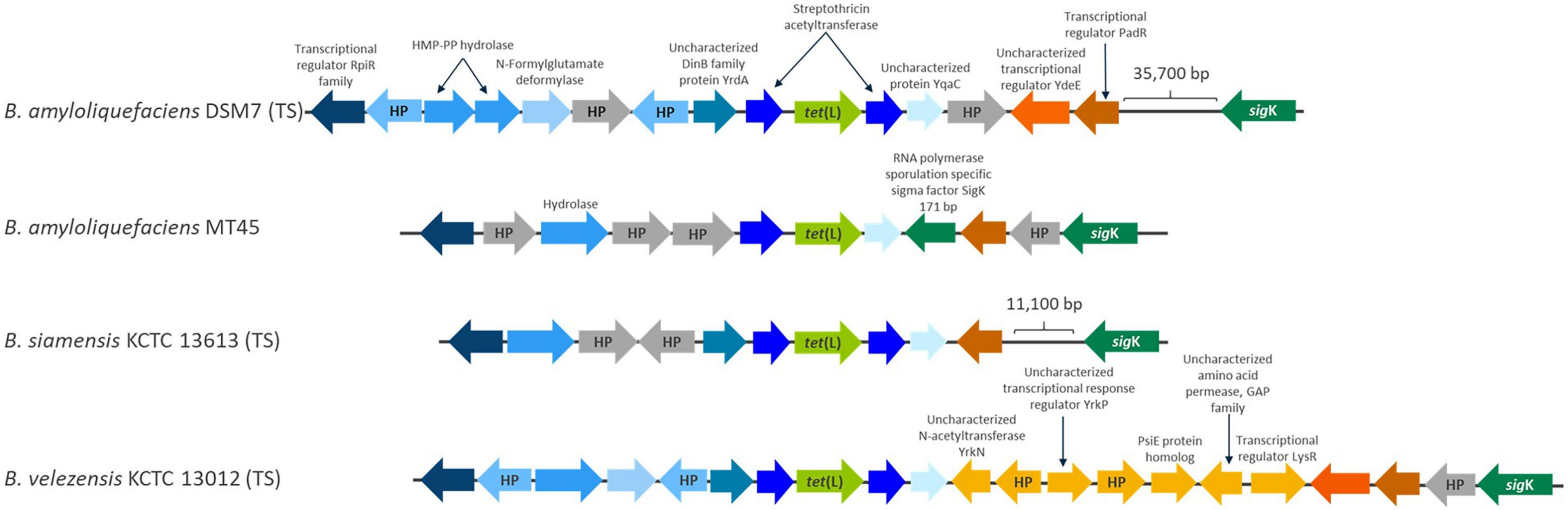
A schematic representation of the genomic position of chromosomally encoded *tet*(L) in *B. amyloliquefaciens* DSM7 (type strain) and MT45, *B. siamensis* KCTC 13613 (type strain) and *Bacillus velezensis* KCTC 13012 (type strain). Genes found in more than one strain are given a blue color and identical genes between strains are the same blue color. HP is used as abbreviation for hypothetical proteins.

The *tet*(L)2.2 genes in *B. amyloliquefaciens, B. velezensis* and *B. siamensis* are positioned in the same genomic region in the three species and flanked by the same genes upstream (Figure 4), which indicates that the *tet*(L)2.2 gene was already present before species differentiation in a common ancestor of the three species. However, some variation occurs within the genomic region downstream of the *tet*(L)2.2 gene and this variation was mainly found between the *tet*(L)2.2 gene and the *sig*K gene (Figure 4), which encode a RNA polymerase sigma factor involved in activation of gene expression during sporulation (Eichenberger et al., 2004). *sig*K is furthermore a known location of inactive prophages, which are often observed in spore-forming bacteria, such as *Bacillus* (Suzuki et al., 2020). The distance between the *tet*(L)2.2 gene and *sig*K varies within and between the different species as different genes have been integrated in the region (Figure 4).

The region in the near vicinity of the *tet*(L)2.2 was inspected for genes associated with mobilization, such as transposons, insertion sequences, plasmids and prophages and revealed that the *B. amyloliquefaciens* strain LMG12263 encodes an incomplete prophage ~40,000 bp downstream of the *tet*(L)2.2 gene as shown through a PHASTER analysis (data not shown). In some of the genomes, genes with phage related annotations was observed in the region between *tet*(L)2.2 and *sig*K, but no intact phages was observed in the vicinity of *tet*(L)2.2.

The genomic position of *tet*(L)2.2 gene in *B. amyloliquefaciens, B. velezensis,* and *B. siamensis* and its flanking genes did not show any resemblance to the flanking genes of the chromosomally and plasmid-encoded *tet*(L)1.1, suggesting that *tet*(L)2.2 have not been acquired through integration of plasmids from other Gram-positive and Gram-negative bacteria.

In general no indications of *tet*(L)2.2 mobilization was found, as the *tet*(L)2.2 gene in *B. amyloliquefaciens, B. velezensis,* and *B. siamensis* are positioned in the same genomic region, are not flanked by intact prophages and form a separate clade in the phylogenetic analysis (Figure 1) that follows the operational group *B. amyloliquefaciens* phylogeny (Figure 3). Rather it seems like *tet*(L)2.2 is stably positioned within operational group *B. amyloliquefaciens* and should therefore be considered an intrinsic resistance gene.

## Conclusion

The tetracycline gene classification system defines a class of genes as being >80% identical at amino acid level and does not consider the phylogenetic relationship. We suggest that further subgrouping based on phylogenetic relationship should be taken into consideration and that the *tet*(L) sub classes should be named *tet*(L)1.1, *tet*(L)1.2 and *tet*(L)2.1, *tet*(L)2.2, corresponding to the four phylogenetic clades observed in the phylogenetic analysis.

We have analyzed the presence of the *tet*(L)2.2 gene (confer low level tetracycline resistance) in the *Bacillus* species belonging to the operational group *B. amyloliquefaciens* (*B. amyloliquefaciens, B. siamensis,* and *B. velezensis*) and our analysis suggest that this gene can be considered intrinsic in these species, although some strains encode a truncated version or lost it over time. This is supported by the high degree of conservation of the gene within the species; the gene being positioned in the same genomic location in the three species, which indicates that it was present already in a common ancestor of the three species before species differentiation, and furthermore that the gene is phylogenetically distinct from *tet*(L)1.1 and *tet*(L)1.2 found on plasmids and chromosomally encoded next to mobile genetic elements in other Gram-positive and Gram-negative bacteria (confer high level tetracycline resistance). The *tet*(L)2.2 from operational group *B. amyloliquefaciens* will therefore with high likelihood not add to the pool of transferable antibiotic resistance genes that can compromise treatments for humans and animals.

Subgrouping of antibiotic resistance genes classes based on phylogenetic relationship will ensure a correct differentiation of intrinsic and acquired antibiotic resistance genes, which is especially important when live bacteria are used for industrial purposes.

## Data availability statement

The datasets presented in this study can be found in online repositories. The names of the repository/repositories and accession number(s) can be found in the article/Supplementary material.

## Author contributions

KN-M wrote the manuscript, made figures, tables, and performed the analysis and was involved in developing the concept and the method and discussed the results. CS was involved in developing the concept, guiding the analysis, discussion, and review and editing. HI was involved in developing the concept, discussion, and review and editing. YA was involved in conceiving the idea, developing and guiding the concept, analysis, design, discussion, and review and editing. All authors contributed to the article and approved the submitted version.

## Funding

This research was funded by Innovation Fund Denmark (grant no. 9065-00029B) as well as internal funding at Chr. Hansen A/S. Chr. Hansen A/S was not involved in the study design, collection, analysis, interpretation of data, the writing of this article or the decision to submit it for publication.

## Conflict of interest

This work was performed by employees of Chr. Hansen A/S, a company that produces strains for plant protection, animal, and human health as well as for the food industry. K-NM, CS, and YA are employees at Chr. Hansen A/S and some are share-holders.

The remaining author declares that the research was conducted in the absence of any commercial or financial relationships that could be construed as a potential conflict of interest.

## Publisher’s note

All claims expressed in this article are solely those of the authors and do not necessarily represent those of their affiliated organizations, or those of the publisher, the editors and the reviewers. Any product that may be evaluated in this article, or claim that may be made by its manufacturer, is not guaranteed or endorsed by the publisher.

## Footnotes

^1^ https://pubmlst.org/organisms/bacillus-subtilis

^2^ https://tygs.dsmz.de/

^3^ http://tree.bio.ed.ac.uk/software/figtree/

^4^ http://cctop.enzim.ttk.mta.hu/?_=
