# Supplementary material for: Intrinsic tet(L) sub-class in Bacillus velezensis and Bacillus amyloliquefaciens is associated with a reduced susceptibility toward tetracycline
Source: Front Microbiol. 2022 Aug 4;13:966016. doi: 10.3389/fmicb.2022.966016 (PMC9387203; doi:10.3389/fmicb.2022.966016)
Supplement: Supplementary file 1 [file Table_1.pdf]

Table S1: Origin of the *B. velezensis* and *B. amyloliquefaciens* included in the study

Strain	Source	Year of isolation	Geographic area	Sequence type (ST)	Genome size (Mb)	GC content (%)	Sequencing method	Assembly level	Number of contigs	Coverage	Reference
B. velezensis											
**KCTC 13012 (Type strain) (LHCC00000000)**	River mouth	2000	Spain	61	4.04	46.30	Illumina HiSeq	Scaffold	NA	100.0x	(16)
**CHBv1 (JAMJPS000000000)**	Isolated from commercial product	≤2015	NA	165	4.13	45.9	NA	Contig	31	NA	(17)
**CHBv2 (JAMJPR00000000)**	Isolated from commercial product	≤2015	NA	42	4.19	45.8	NA	Contig	26	NA	(17)
**CHBv3 (JAMJPQ00000000)**	Isolated from commercial product	≤2015	NA	175	3.99	46.3	NA	Contig	29	NA	(17)
**LMG12384 (JAMJPP000000000)**	Cocoa	≤2016	Malaysia	167	3.86	46.6	NA	Contig	18	NA	(17)
**CHCC26801 (SAMN08399260)**	Water	1999	Spain	168	4.01	46.3	Illumina MiSeq	Contig	20	40.0x	(17)
**CBMB205 (CP011937)**	Rice rhizosphere soil	2004	South Korea	96	3.93	46.5	PacBio	Complete genome	NA	194.0x	NCBI
**CGMCC 11640 (CP026610)**	Bamboo forest soil	2013	China	247	4.39	45.62	Illumina, PacBio	Complete genome	NA	91.0x	NCBI
**DSYZ (CP030150)**	Rhizosphere soil	2012	China	247	4.32	45.72	Illumina MiSeq, PacBio	Complete genome	NA	425.0x	(18)
**Lzh-a42 (CP025308)**	Tomato rhizosphere soil	2015	China	61	4.25	46.00	Illumina MiSeq, PacBio	Complete genome	NA	278.0x	(19)
**W1 (CP028375)**	Two-spotted spider mites	2017	China	61	4.24	45.90	Illumina MiSeq, PacBio	Complete genome	NA	328.0x	(20)
**CHBv6 (CP025079)**	Isolated from commercial product	2015	France	42	4.23	45.90	Illumina HiSeq	Complete genome	NA	100.0x	(21)
**Bac57 (CP033054)**	Red Sea Lagoons-Mangrove Mud	2012	Saudi Arabia	91	4.23	45.89	PacBio	Complete genome	NA	344.0x	(22)
**9912D (CP017775)**	Sediment sample from the Liaodong Bay of the Bohai Sea	1998	China	110	4.24	45.96	PacBio, Illumina HiSeq	Complete genome	NA	350.0x	(23)
**DKU_NT_04 (CP026533)**	Fermented soybean	2017	South Korea	68	4.33	45.23	PacBio	Complete genome	NA	192.0x	NCBI
**SCDB 291 (CP022654)**	Traditional fermented soybean paste (Doenjang)	2012	South Korea	110/158	4.16	46.40	PacBio	Complete genome	NA	237.0x	NCBI
**AGVL-005 (CP024922)**	Soybean seeds	2015	Brazil	163	4.15	46.00	Minion	Complete genome	NA	160.0x	(24)
**CC09 (CP015443)**	Cinnamomum camphora leaves	2007	China	247	4.17	46.10	Illumina MiSeq	Complete genome	NA	100.0x	(25)
**1B-23 (CP033967)**	Potato rhizosphere	2012	Canada	70	4.14	46.10	PacBio	Complete genome	NA	100.3x	NCBI
**SRCM102747 (CP028211)**	Kimchi	2017	South Korea	64/70/115/149/163	4.10	46.20	PacBio	Complete genome	NA	336.0x	NCBI
**SRCM101368 (CP031694)**	Ganjang (Korean Soy Sauce)	2016	South Korea	95/135	4.09	46.30	PacBio RSII	Complete genome	NA	239.0x	NCBI

**S4 (CP050424)**	Agricultural soil	2011	USA	41/39/63/163	4.07	46.40	PacBio RSII	Complete genome	NA	315.0x	(26)
**EN01 (CP053377)**	Leaf	2018	Taiwan	71	4.03	46.50	PacBio Sequel	Complete genome	NA	785.6x	NCBI
**JS25R (CP009679)**	Wheat	2012	China	66	4.01	46.39	Illumina	Complete genome	NA	256.0x	NCBI
**83 (CP034203)**	Mango Orchard	2000	Mexico	91	4.00	46.40	Illumina MiSeq	Complete genome	NA	400.0x	NCBI
**CN026 (CP024897)**	Chicken feces	2014	Belgium	140	4.00	46.40	Illumina MiSeq	Complete genome	NA	606.0x	(27)
**G341 (CP011686)**	4-year-old roots of Korean ginseng	2012	South Korea	132	4.01	46.50	454	Complete genome	NA	80.0x	(28)
**UCMB5007 (CP041143)**	Bull calf intestinal microflora	1989	Ukraine	40/133/161/247	3.98	46.60	Illumina MiSeq, Illumina HiSeq, PacBio RSII	Complete genome	NA	1000.0x	(29)
**UCMB5044 (CP041144)**	Inner tissues of cotton plant (Gossypium sp.)	1990	Tajikistan	40/133/161/247	3.98	46.60	Illumina MiSeq, Illumina HiSeq, PacBio RSII	Complete genome	NA	1000.0x	(29)
**UCMB5140 (CP051463)**	Therapeutic muds	1987	Estonia	63	3.98	46.50	PacBio RSII	Complete genome	NA	150x	NCBI
**BIM B-4390D (CP032144)**	Soil	2005	Belarus	93	3.98	46.50	Illumina MiSeq, Sanger	Complete genome	NA	150.0x	NCBI
**BvL03 (CP041192)**	Sediment samples of fishpond	2017	China	68	3.98	46.60	PacBio	Complete genome	NA	867.0x	(30)
**GYL4 (CP020874)**	Pepper plant	2015	Korea	69	3.98	46.50	PacBio	Complete genome	NA	259.0x	NCBI
**S141 (AP018402)**	Rhizosphere	2009	Thailand	111	3.97	46.50	Illumina MiSeq	Complete genome	NA	717.0x	(31)
**ZF2 (CP032154)**	Cucumber plant	2017	China	96	3.93	46.50	PacBio Sequel	Complete genome	NA	510.0x	(32)
**S3-1 (CP016371)**	Cucumber rhizosphere	2012	China	96	3.93	46.50	Illumina MiSeq, PacBio	Complete genome	NA	240.0x	NCBI
**GQJK49 (CP021495)**	Lycium barbarum rhizosphere	2016	China	96	3.93	46.50	PacBio	Complete genome	NA	251.0x	(33)
**ANSB01E (CP036518)**	Chicken intestine	2012	China	96	3.93	46.50	PacBio PSII	Complete genome	NA	616.0x	(34)
**JT3-1 (CP032506)**	Yak feces	2017	China	96	3.93	46.50	PacBio Sequel	Complete genome	NA	100.0x	(35)
**DH8043 (CP047268)**	Oyster	2019	China	61/66/91/140/169	3.97	46.20	Nanopore	Complete genome	NA	166.51x	NCBI
**LDO2 (CP029034)**	Peanut root	2017	China	96	3.95	46.50	PacBio	Complete genome	NA	329.0x	NCBI
**BIM B-1312D (CP050448)**	Soil	2018	Belarus	96	3.93	46.50	Illumina MiSeq, Oxford Nanopore MinIION	Complete genome	NA	400.0x	NCBI
**UB2017 (CP049741)**	Citrus rhizosphere	2017	China	96	3.93	46.50	Oxford Nanopore PromethION, MiSeq	Complete genome	NA	176.0x	NCBI
**CMT-6 (CP025341)**	Chinese traditional food Douchi	2012	China	135/174	3.93	46.50	Illumina HiSeq, PacBio	Complete genome	NA	200.0x	(36)
**ZL918 (CP021338)**	Sagittaria sagittifolia infected bulbs	2013	China	94	3.92	46.50	Illumina MiSeq	Complete genome	NA	100.0x	(37)
**LS69 (CP015911)**	Rice field	2015	China	96	3.92	46.50	Illumina	Complete genome	NA	256.0x	NCBI

**JTYP2 (CP020375)**	Leaf surface	2015	China	96	3.93	46.50	PacBio	Complete genome	NA	980.0x	(38)
**At1 (CP041145)**	Arabidopsis thaliana seedling from surface sterilized seed	2000	Sweden	65	3.89	46.70	Illumina MiSeq, PacBio Sequel	Complete genome	NA	500.0x	(39)
**Hx05 (CP029473)**	Banana rhizosphere soil	2010	China	95/99	3.91	46.50	PacBio	Complete genome	NA	500.0x	NCBI
**CHBv4 (JAMJPO00000000)**	Isolated from commercial product	≤2015	NA	164	3.87	46.50	NA	Contig	26	NA	(17)
**CHBv5 (JAMJPN00000000)**	Isolated from commercial product	≤2015	NA	93	3.90	45.50	NA	Contig	38	NA	(17)
**FS1092 (CP038028)**	Food processing environment	2016	NA	42	4.24	45.90	Illumina MiSeq, Oxford Nanopore MiniIon	Complete genome	NA	58.0x	(40)
**MBE1283 (CP013727)**	Korean traditional alcoholic beverage	2014	South Korea	60/99	3.98	46.49	PacBio	Complete genome	NA	128.0x	NCBI
**Y14 (CP017953)**	Peanut rizosphere	2013	China	99	3.96	46.40	PacBio, Illumina HiSeq	Complete genome	NA	155.0x	NCBI
**KHG19 (CP007242)**	Korean traditional fermented soybean paste, Doenjang	2012	South Korea	148	3.95	46.60	454, Illumina	Complete genome	NA	38.0x	NCBI
**X030 (CP040672)**	Peanut	2014	China	Cannot be determined	3.95	46.60	Illumina HiSeq	Complete genome	NA	85.76x	(41)
**WS-8 (CP018200)**	Soil from hillside	2013	China	96	3.93	46.50	PacBio	Complete genome	NA	311.0x	NCBI
**S499 (CP014700)**	Plant soil	1952	Democratic Republic of Congo	60	3.94	46.59	PacBio RSII	Complete genome	NA	78.0x	NCBI
**V417 (CP044359)**	Grapefruit	NA	NA	41	3.91	46.50	454, Oxford Nanopore	Complete genome	NA	70.0x	NCBI
**WF02 (CP053376)**	Soil	2012	Taiwan	71	4.03	46.50	Illumina MiSeq, PacBio RSIi	Complete genome	NA	153.7x	NCBI
**ARP23 (CP035899)**	Wheatgrass	2000	Argentina	NA	4.02	46.50	PacBio	Complete genome	NA	224.0x	NCBI
**B15 (CP014783)**	Grape skin	2015	China	247	4.01	46.50	Illumina	Complete genome	NA	160.0x	(42)
**DH8030 (CP041770)**	Oyster	2019	China	71	3.99	46.50	Nanopore	Complete genome	NA	306.19x	NCBI
**LM2303 (CP018152)**	Wild yak feces	2010	China	NA	3.99	46.70	PacBio	Complete genome	NA	239.0x	NCBI
**ALB79 (CP029071)**	Grape	2016	USA	42	3.98	46.40	PacBio, Illumina MiSeq	Complete genome	NA	305.0x	(43)
**Y2 (CP003332)**	Wheat rhizosphere	NA	NA	61	4.24	45.90	Illumina	Complete genome	NA	NA	(44)
**KC41 (CP044444)**	Soil	2019	South Korea	68/91/110/172	4.12	46.00	PacBio RSIi	Complete genome	NA	227.0x	NCBI
**ZJU1 (CP041691)**	Morbus alba	2017	China	61	4.96	46.40	PacBio	Complete genome	NA	284.0x	(45)
**ALB69 (CP029070)**	Almond drupe	2011	USA	61	4.05	46.50	PacBio, Illumina MiSeq	Complete genome	NA	232.0x	(43)
**SH-B74 (CP030097)**	Marine sediment	2003	China	32/64/93/132/137	4.10	46.41	PacBio	Complete genome	NA	100.0x	NCBI

**ALB65 (CP029069)**	Alfalfa silage	2011	USA	91	4.04	46.40	Illumina MiSeq, PacBio	Complete genome	NA	211.0x	(43)
**UMAF6639 (CP006058)**	NA	NA	NA	115	4.03	46.30	Illumina, Solexa	Complete genome	NA	100.0x	(46)
B. amyloliquefaciens											
**DSM 7 (Type strain) (FN597644)**	NA	NA	NA	117	3.98	46.10	NA	Complete genome	NA	NA	(47)
**CHBa1 (SAMN28557232)**	Pig feces from slaughter pigs, not receiving antibiotics or Bacillus based products	≤2011	Germany	59	3.96	46.10	NA	Contig	58	NA	(17)
**LMG12263 (JAMJPM00000000)**	NA	≤2012	NA	117	3.99	45.60	NA	Contig	35	NA	(17)
**H (CP041693)**	NA	1975	USA	59	3.95	45.90	PacBio RSII	Complete genome	NA	71.49x	NCBI
**MT45 (CP011252)**	Daqu	2014	China	178	3.90	46.10	Illumina	Complete genome	NA	293.0x	(48)
**RD7-7 (CP016913)**	Fermented soybean paste	2015	South Korea	179	3.69	46.30	PacBio	Complete genome	NA	231.0x	NCBI
**YP6 (CP032146)**	Rhizosphere of Lolium perenne	NA	China	177	4.01	45.90	PacBio, Illumina HiSeq	Complete genome	NA	256.0x	(49)
**Ba13 (CP073635)**	Soil	2017	China	171	3.86	46.2	Illumina, Oxford Nanopore	Complete genome	NA	300.0x	NCBI
**LL3 (CP002634)**	Fermented food (Korean bibimbab)	≤2011	NA	NA	3.99	45.7	NA	Complete genome	NA	70x	NCBI
**TA208 (CP002627)**	NA	≤2011	NA	59	3.94	45.8	NA		NA	106.4x	NCBI
**HK1 (CP018902)**	Corn stalk residue compost	2007	China	117	4.00	46.00	SMRT PacBio	Complete genome		200.0x	NCBI
**SRCM101267 (CP021505)**	Food	2017	South Korea	117	4.09	45.89	PacBio	Complete genome		350.0x	NCBI
**205 (CP054415)**	Soil	2015	China	117			PacBio	Complete genome		300.0x	NCBI
B. siamensis											
**KCTC 13613 (Type strain) (AJVF01000002)**	NA	≤2013	NA	NA	3.78	46.30	Illumina HiSeq	Contigs	51	500.0x	(52)
**7551 (NPCI01000021)**	Uncut heroin sample	≤2017	NA	NA	3.72	46.40	Illumina HiSeq	Contig	67	172.14x	(53)
**B28 (CP066219)**	Galchi-Kimchi	2016	South korea	NA	3.96	45.89	PacBio, Miseq	Complete genome	NA	-	(54)
**JJC33M (JTJG01000003)**	Soil	2010	Mexico	NA	3.96	45.70	Illumina HiSeq	Contigs	40	240.0x	(55)
**SCSIO 05746 (CP025001)**	Sea mud (sediment)	2012	Indian Ocea	NA	4.28	45.98	Illumina HiSeq, PacBio	Complete genome	NA	360.0x	(37)
**SRCM100169 (LYUE01000001)**	Kochujang	2011	South Korea	NA	3.97	45.80	Illumina MiSeq	Contigs	45	293.0x	NCBI
**XY18 (LAGT01000048)**	Cured vanilla bean	2014	China	NA	4.02	46.40	IonTorrent	Contigs	63	52.0x	(56)

≤ in front of the year indicates that only the date of deposit is known, NA: not available

Tabel S2: *tet*(L) encoded by Gram-positive and -negative bacteria.

Strains	Genomic position	Accession number	Length (bp)	GC content (%)	Start codon	Predicted transmembrane domains	MIC tested	Reference
**Gram-positive bacteria**								
Bacillus cereus	Plasmid (pBC16)	X51366	1377	35.37	GTG	14		(1)
Bacillus cereus AL1	Plasmid (pJHI)	AY129652	1377	35.37	GTG	14		NCBI
Bacillus stearothermophilus T15	Plasmid (pTHT15)	M11036	1377	35.29	GTG	14		(2)
Bacillus subtilis R	Chromosome	D12567	1377	40.23	GTG	14		(3)
Bacillus subtilis 168	Chromosome	AL009126	1377	39.94	GTG	14		(4)
Bacillus subtilis NCIB 3610	Chromosome	CP020102	1377	39.9	GTG	14		NCBI
Bacillus subtilis 6051-HGW	Chromosome	CP003329	1377	39.9	GTG	14		(5)
Bacillus subtilis NBRC 13719	Chromosome	AP019714	1377	39.9	GTG	14		NCBI
Bacillus subtilis MB9_B1	Chromosome	CP045820	1377	40.1	GTG	14		(6)
Bacillus subtilis BS49Ch	Chromosome	LN649259	1377	39.9	GTG	14		NCBI
Bacillus subtilis HJ0-6	Chromosome	CP016894	1377	39.9	GTG	14		NCBI
Bacillus subtilis BS34A	Chromosome	LN680001	1377	39.9	GTG	14		NCBI
Bacillus subtilis SRCM103612	Chromosome	CP035406	1377	39.6	GTG	14		NCBI
Bacillus subtilis MB8_B10	Chromosome	CP045824	1377	39.9	GTG	14		(6)
Bacillus subtilis MB8_B1	Chromosome	CP045823	1377	40.1	GTG	14		(6)
Bacillus subtilis JAAA	Chromosome	CP045425	1377	39.6	GTG	14		NCBI
Bacillus subtilis	Plasmid (pNS1981)	D00006	1377	35.29	GTG	14		(2)
Bacillus sp. 24	Plasmid (pBHS24)	HM235948	1383	35.14	GTG	14		(7)
Enterococcus durans VREdu	Plasmid (pSULI)	CP043327	1377	35.37	GTG	14		NCBI
Enterococcus faecalis Jh1	Plasmid (pJH1)	U17153	1377	35.37	GTG	14		(8)
Enterococcus faecium	NA	AY081910	1377	35.29	GTG	14		(9)
Enterococcus faecium KN9	Transposon	KP036966	1287	35.90	ATG	13		NCBI
Limosilactobacillus reuteri ZLR003	Chromosome	CP014786	1377	35.22	GTG	14		NCBI
Latilactobacillus sakei Rits9	Plasmid (pLS55)	EF605268	1383	35.1	ATG	14		(10)
Paenibacillus larvae	Plasmid (pMA67)	DQ367664	1383	35.2	GTG	14		(11)
Staphylococcus aureus B9-22D	Plasmid (pSALNP9)	CP042083	1377	35.37	GTG	14		NCBI
Staphylococcus aureus NX-T55	Chromosome	CP031839	1377	35.37	GTG	14		NCBI
Staphylococcus aureus ST398	Plasmid (pKKS825)	FN377602	1380	35.36	ATG	14		(12)
Staphylococcus hyicus Shy 17-pSTE1	Plasmid (pSTE1)	X60828	1377	35.66	GTG	14		(13)
Streptococcus agalactiae	Plasmid (pLS1)	M29725	1377	35.37	GTG	14		(14)
Streptococcus suis 74911-8	Chromosome	KY400493	1203	35.74	ATG	12		NCBI
**Gram-negative bacteria**								
Campylobacter jejuni ZJB020	Chromosome	CP048769	1377	35.29	GTG	14		NCBI (15)
Campylobacter jejuni ZS004	Plasmid (pCJFEX)	CP048762	1377	35.29	GTG	14		NCBI (15)
Bacillus velezensis								

KCTC 13012 (Type strain)	Chromosome	LHCC00000000.1	1377	43.65	GTG	14		(16)
CHBv1	Chromosome	JAMJPS000000000	1377	43.35	GTG	14	Yes	(17)
CHBv2	Chromosome	JAMJPR000000000	1377	43.06	GTG	14	Yes	(17)
CHBv3	Chromosome	JAMJPQ000000000	1377	43.36	GTG	14	Yes	(17)
LMG12384	Chromosome	JAMJPP000000000	1377	43.21	GTG	14	Yes	(17)
CHCC26801	Chromosome	SAMN08399260	1377	43.72	GTG	14	Yes	(17)
CBMB205	Chromosome	CP011937	1377	43.43	GTG	14		NCBI
CGMCC 11640	Chromosome	CP026610	1377	43.43	GTG	14		NCBI
DSYZ	Chromosome	CP030150	1377	43.43	GTG	14		(18)
Lzh-a42	Chromosome	CP025308	1377	43.65	GTG	14		(19)
W1	Chromosome	CP028375	1377	43.65	GTG	14		(20)
CHBv6	Chromosome	CP025079	1377	43.06	GTG	14		(21)
Bac57	Chromosome	CP033054	1377	43.50	GTG	14		(22)
9912D	Chromosome	CP017775	1377	43.57	GTG	14		(23)
DKU_NT_04	Chromosome	CP026533	1377	43.14	GTG	14		NCBI
SCDB 291	Chromosome	CP022654	1377	43.43	GTG	14		NCBI
AGVL-005	Chromosome	CP024922	-	-	GTG	14		(24)
CC09	Chromosome	CP015443	1377	43.43	GTG	14		(25)
1B-23	Chromosome	CP033967	498	43.17	GTG	14		NCBI
SRCM102747	Chromosome	CP028211	402/222	44.78/42.34	GTG	14		NCBI
SRCM101368	Chromosome	CP031694	1377	43.57	GTG	14		NCBI
S4	Chromosome	CP050424	1377	43.65	GTG	14		(26)
EN01	Chromosome	CP053377	1377	43.28	GTG	14		NCBI
JS25R	Chromosome	CP009679	1377	43.94	GTG	14		NCBI
83	Chromosome	CP034203	1377	43.94	GTG	14		NCBI
CN026	Chromosome	CP024897	1377	44.01	GTG	14		(27)
G341	Chromosome	CP011686	1377	43.79	GTG	14		(28)
UCMB5007	Chromosome	CP007242	1377	43.86	GTG	14		(29)
UCMB5044	Chromosome	CP041144	1377	43.86	GTG	14		(29)
UCMB5140	Chromosome	CP051463	1377	43.43	GTG	14		NCBI
BIM B-4390D	Chromosome	CP032144	1377	43.65	GTG	14		NCBI
BvL03	Chromosome	CP041192	1377	43.43	GTG	14		(30)
GYL4	Chromosome	CP020874	1377	44.44	GTG	14		NCBI
S141	Chromosome	AP018402	345	41.74	GTG	14		(31)
ZF2	Chromosome	CP032154	1377	43.43	GTG	14		(32)
S3-1	Chromosome	CP016371	1377	43.43	GTG	14		NCBI
GQJK49	Chromosome	CP021495	1377	43.43	GTG	14		(33)
ANSB01E	Chromosome	CP036518	1377	43.43	GTG	14		(34)
JT3-1	Chromosome	CP032506	1377	43.43	GTG	14		(35)
DH8043	Chromosome	CP047268	1377	43.72	GTG	14		NCBI
LDO2	Chromosome	CP029034	1377	43.43	GTG	14		NCBI
BIM B-1312D	Chromosome	CP050448	1377	43.43	GTG	14		NCBI
UB2017	Chromosome	CP049741	1377	43.43	GTG	14		NCBI

CMT-6	Chromosome	CP025341	1377	43.50	GTG	14		(36)
ZL918	Chromosome	CP021338	1377	43.43	GTG	14		(37)
LS69	Chromosome	CP015911	1377	43.43	GTG	14		NCBI
JTYP2	Chromosome	CP020375	1377	43.43	GTG	14		(38)
At1	Chromosome	CP041145	1377	43.72	GTG	14		(39)
Hx05	Chromosome	CP029473	1377	43.21	GTG	14		NCBI
CHBv4	Chromosome	JAMJPO000000000	1377	43.94	GTG	14	Yes	(17)
CHBv5	Chromosome	JAMJPN000000000	498	43.17	GTG	14	Yes	(17)
FS1092	Chromosome	CP038028	1377	43.06	GTG	14		(40)
MBE1283	Chromosome	CP013727	1374	43.45	GTG	14		NCBI
Y14	Chromosome	CP017953	1377	43.21	GTG	14		NCBI
KHG19	Chromosome	CP007242	1377	43.65	GTG	14		NCBI
X030	Chromosome	CP040672	1377	43.43	GTG	14		(41)
WS-8	Chromosome	CP018200	1377	43.43	GTG	14		NCBI
S499	Chromosome	CP014700	1377	43.36	GTG	14		NCBI
V417	Chromosome	CP044359	-	-	GTG	14		NCBI
WF02	Chromosome	CP053376	1377	43.28	GTG	14		NCBI
ARP23	Chromosome	CP035899	1032/327	43.31/41.90	GTG	14		NCBI
B15	Chromosome	CP014783	711	42.76	GTG	14		(42)
DH8030	Chromosome	CP041770	1377	43.28	GTG	14		NCBI
LM2303	Chromosome	CP018152	1377	43.50	GTG	14		NCBI
ALB79	Chromosome	CP029071	1377	43.06	GTG	14		(43)
Y2	Chromosome	CP003332	1377	43.65	GTG	14		(44)
KC41	Chromosome	CP044444	1377	43.50	GTG	14		NCBI
ZJU1	Chromosome	CP041691	1377	43.65	GTG	14		(45)
ALB69	Chromosome	CP029070	1377	43.57	GTG	14		(43)
SH-B74	Chromosome	CP030097	1377	43.94	GTG	14		NCBI
ALB65	Chromosome	CP029069	1377	43.79	GTG	14		(43)
UMAF6639	Chromosome	CP006058	1377	43.36	GTG	14		(46)
Bacillus amyloliquefaciens								
DSM 7 (Type strain)	Chromosome	FN597644	1377	43.79	GTG	14		(47)
CHBa1	Chromosome	SAMN28557232	1377	42.63	GTG	14	Yes	(17)
LMG12263	Chromosome	JAMJPM000000000	1377	43.72	GTG	14	Yes	(17)
H	Chromosome	CP041693	1377	42.63	GTG	14		NCBI
MT45	Chromosome	CP011252	1377	42.70	GTG	14		(48)
RD7-7	Chromosome	CP016913	315	44.76	-	-		NCBI
YP6	Chromosome	CP032146	1377	42.34	GTG	14		(49)
Ba13	Chromosome	CP073635	-	-	-	-		NCBI
LL3	Chromosome	CP002634	1377	42.60	GTG	14		(50)
TA208	Chromosome	CP002627	1377	42.60	GTG	14		(51)
HK1	Chromosome	CP018902	1377	43.80	GTG	14		NCBI
SRCM101267	Chromosome	CP021505	1377	43.80	GTG	14		NCBI
205	Chromosome	CP054415	1377	43.80	GTG	14		NCBI

Bacillus siamensis							
KCTC 13613 (Type strain)	Chromosome	AJVF01000002	1377	43.50	GTG	14	NCBI
7551	Chromosome	NPCI01000021	1377	43.72	GTG	14	NCBI
B28	Chromosome	CP066219	1377	43.28	GTG	14	NCBI
JJC33M	Chromosome	JTJG01000003	1377	43.57	GTG	14	NCBI
SCSIO 05746	Chromosome	CP025001	1377	43.06	GTG	14	NCBI
SRCM100169	Chromosome	LYUE01000001	1377	43.65	GTG	14	NCBI
XY18	Chromosome	LAGT01000048	1377	43.57	GTG	14	NCBI

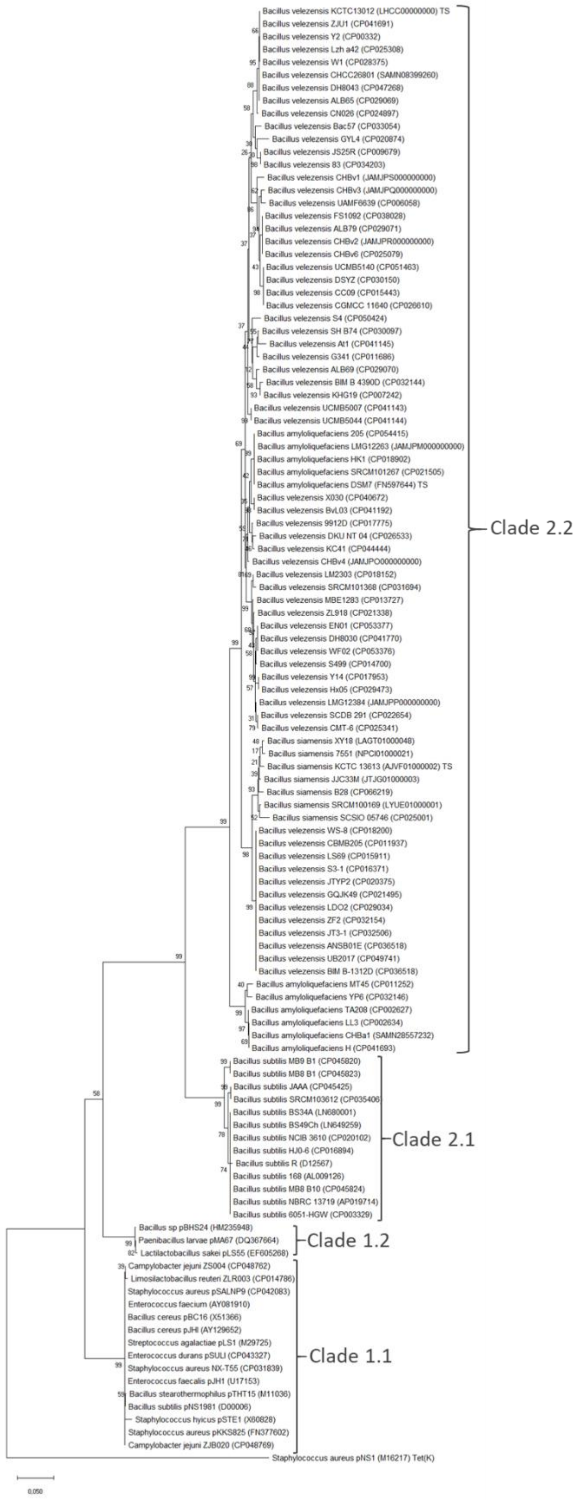

Figure S1. Phylogenetic tree of the *tet*(L) gene. The tree was built by evolutionary analysis by Maximum Likelihood method and Tamura-Nei model by MEGA X (57,58). The branch lengths are measured in the number of substitutions per site. Strain name and GenBank accession number is provided for the sequences. The phylogenetic trees were rooted with *Staphylococcus aureus tet*(L) (M16217) as an outgroup. The numbers at the nodes are bootstrap values (1-100).

1. Palva A, Vidgren G, Simonen M, Rintala H, Laamanen P. Nucleotide sequence of the tetracycline resistance gene of pBC16 from Bacillus cereus. Nucleic Acids Res. 1990;18(6):1635.

2. Takayuki H, Takayuki I, Noboru T, Kensuke F. Nucleotide sequence of the tetracycline resistance gene of pTHT15, a thermophilic Bacillus plasmid: comparison with staphylococcal TcR controls. Gene. 1985;37(1–3):131–8.

3. Amano H, Saito Y, Shishido K. The nucleotide sequence of the chromosomal tetracycline-resistance gene of Bacillus subtilis R differs from that of B. Subtilis 168 by five base pairs. J Gen Appl Microbiol. 1993;39(3):321–6.

4. Sakaguchi R, Amano H, Shishido K. Nucleotide sequence homology of the tetracycline-resistance determinant naturally maintained in Bacillus subtilis Marburg 168 chromosome and the tetracycline-resistance gene of B. subtilis plasmid pNS1981. BBA - Gene Struct Expr. 1988;950(3):441–4.

5. Kabisch J, Thürmer A, Hübel T, Popper L, Daniel R, Schweder T. Characterization and optimization of Bacillus subtilis ATCC 6051 as an expression host. J Biotechnol [Internet]. 2013;163(2):97–104. Available from: http://dx.doi.org/10.1016/j.jbiotec.2012.06.034

6. Kiesewalter HT, Lozano-Andrade CN, Maróti G, Snyder D, Cooper VS, Jørgensen TS, et al. Complete Genome Sequences of 13 Bacillus subtilis Soil Isolates for Studying Secondary Metabolite Diversity. Microbiol Resour Announc. 2020;9(2):6–8.

7. Phelan RW, Clarke C, Morrissey JP, Dobson ADW, O'Gara F, Barbosa TM. Tetracycline resistance-encoding plasmid from Bacillus sp. strain #24, isolated from the marine sponge haliclona simulans. Appl Environ Microbiol. 2011;77(1):327–9.

8. Platteeuw C, Michiels F, Joos H, Seurinck J, de Vos WM. Characterization and heterologous expression of the tetL gene and identification of iso-ISS1 elements from Enterococcus faecalis plasmid pJH1. Gene. 1995;160(1):89–93.

9. G. W, RJ. W, B. H, I. K, W. W. Influence of transferable genetic determinants on the outcome of typing methods commonly used for Enterococcus faecium. J Clin Microbiol [Internet]. 2003;41(4 PG-1499–506):1499–506. Available from: NS -

10. Ammor MS, Gueimonde M, Danielsen M, Zagorec M, Van Hoek AHAM, De Los Reyes-Gavilán CG, et al. Two different tetracycline resistance mechanisms, plasmid-carried tet(L) and chromosomally located transposon-associated tet(M), coexist in Lactobacillus sakei rits 9. Appl Environ Microbiol. 2008;74(5):1394–401.

11. Murray KD, Aronstein KA. Oxytetracycline-resistance in the honey bee pathogen paenibacillus larvae is encoded on novel plasmid pMA67. J Apic Res. 2006;45(4):207–14.

12. Kadlec K, Schwarz S. Novel ABC transporter gene, vga(C), located on a multiresistance plasmid from a porcine methicillin-resistant Staphylococcus aureus ST398 strain. Antimicrob Agents Chemother. 2009;53(8):3589–91.

13. Schwarz S, Cardoso M, Wegener HC. Nucleotide sequence and phylogeny of the tet(L) tetracycline resistance determinant encoded by plasmid pSTE1 from Staphylococcus hyicus. Antimicrob Agents Chemother. 1992;36(3):580–8.

14. Lacks SA, Lopez P, Greenberg B, Espinosa M. Identification and analysis of genes for tetracycline resistance and replication functions in the broad-host-range plasmid pLS1. J Mol Biol. 1986;192(4):753–65.

15. Tang B, Tang B, Tang Y, Tang Y, Zhang L, Zhang L, et al. Emergence of fexA in mediating resistance to florfenicols in campylobacter. Antimicrob Agents Chemother. 2020;64(7):1–8.

16. Jeong H, Park SH, Choi SK. Genome sequence of antibiotic-producing Bacillus amyloliquefaciens strain KCTC 13012. Genome Announc. 2015;3(5):5–6.

17. Agersø Y, Stuer-Lauridsen B, Bjerre K, Jensen MG, Johansen E, Bennedsen M, et al. Antimicrobial susceptibility

testing and tentative epidemiological cutoff values for five Bacillus species relevant for use as animal feed additives or for plant protection. Appl Environ Microbiol. 2018;84(19):1–12.

18. Zhao J, Liu H, Liu K, Li H, Peng Y, Liu J, et al. Complete Genome Sequence of Bacillus velezensis DSYZ, a Plant Growth-Promoting Rhizobacterium with Antifungal Properties . Microbiol Resour Announc. 2019;8(8):21–3.

19. Li Z, Chen M, Ran K, Wang J, Zeng Q, Song F. Draft genome sequence of Bacillus velezensis Lzh-a42, a plant growthpromoting rhizobacterium isolated from tomato rhizosphere. Genome Announc. 2018;6(12):42–3.

20. Li XY, Munir S, Cui WY, He PJ, Yang J, He PF, et al. Genome sequence of Bacillus velezensis W1, a strain with strong acaricidal activity against two-spotted spider mite (Tetranychus urticae). Appl Ecol Environ Res. 2019;17(2):2689–99.

21. Pandin C, Le Coq D, Deschamps J, Védie R, Rousseau T, Aymerich S, et al. Complete genome sequence of Bacillus velezensis QST713: A biocontrol agent that protects Agaricus bisporus crops against the green mould disease. J Biotechnol [Internet]. 2018;278(April):10–9. Available from: https://doi.org/10.1016/j.jbiotec.2018.04.014

22. Othoum G, Prigent S, Derouiche A, Shi L, Bokhari A, Alamoudi S, et al. Comparative genomics study reveals Red Sea Bacillus with characteristics associated with potential microbial cell factories (MCFs). Sci Rep. 2019;9(1):1–13.

23. Pan HQ, Li QL, Hu JC. The complete genome sequence of Bacillus velezensis 9912D reveals its biocontrol mechanism as a novel commercial biological fungicide agent. J Biotechnol [Internet]. 2017;247:25–8. Available from: http://dx.doi.org/10.1016/j.jbiotec.2017.02.022

24. Pylro VS, Dias ACF, Andreote FD, Morais DK, de Mello Varani A, Andreote CCF, et al. Closed genome sequence of phytopathogen biocontrol agent bacillus velezensis strain AGVL-005, isolated from soybean. Genome Announc. 2018;6(7):5–6.

25. Cai X, Kang X, Xi H, Liu C, Xue Y. Complete genome sequence of the endophytic biocontrol strain Bacillus velezensis CC09. Genome Announc. 2016;4(5):2015–6.

26. Hempel PP, Yao M, Yannarell S, Shevchenko O, Vogt F, Donofrio N, et al. Complete Genome Sequence of Bacillus velezensis Strain S4, Isolated from Biochar-Treated Soil . Microbiol Resour Announc. 2020;9(20):3–4.

27. Nannan C, Gillis A, Caulier S, Mahillon J. Complete genome sequence of Bacillus velezensis CN026 exhibiting antagonistic activity against Gram-negative foodborne pathogens. Genome Announc. 2018;6(4):1–2.

28. Lee HH, Park J, Lim JY, Kim H, Choi GJ, Kim JC, et al. Complete genome sequence of Bacillus velezensis G341, a strain with a broad inhibitory spectrum against plant pathogens. J Biotechnol [Internet]. 2015;211:97–8. Available from: http://dx.doi.org/10.1016/j.jbiotec.2015.07.005

29. Reva ON, Swanevelder DZH, Mwita LA, Mwakilili AD, Muzondiwa D, Joubert M, et al. Genetic, Epigenetic and Phenotypic Diversity of Four Bacillus velezensis Strains Used for Plant Protection or as Probiotics. Front Microbiol. 2019;10(November):1–25.

30. Cao L, Pan L, Gong L, Yang Y, He H, Li Y, et al. Interaction of a novel Bacillus velezensis (BvL03) against Aeromonas hydrophila in vitro and in vivo in grass carp. Appl Microbiol Biotechnol. 2019;103(21–22):8987–99.

31. Sibponkrung S, Kondo T, Tanaka K, Tittabutr P, Boonkerd N, Teaumroong N, et al. Genome sequence of Bacillus velezensis S141, a new strain of plant growthpromoting rhizobacterium isolated from soybean rhizosphere. Genome Announc. 2017;5(48):7–8.

32. Xu S, Xie X, Zhao Y, Shi Y, Chai A, Li L, et al. Whole-genome analysis of bacillus velezensis ZF2, a biocontrol agent that protects cucumis sativus against corynespora leaf spot diseases. 3 Biotech [Internet]. 2020;10(4):1–14. Available from: https://doi.org/10.1007/s13205-020-2165-y

33. Ma J, Liu H, Wang C, Li Y, Hou Q, Yao L, et al. Complete Genome Sequence of Bacillus velezensis GQJK49, a Plant GrowthPromoting Rhizobacterium with Antifungal Activity. 2017;5(35):1–2.

34. Guo Y, Zhou J, Tang Y, Ma Q, Zhang J, Ji C, et al. Characterization and Genome Analysis of a Zearalenone-Degrading Bacillus velezensis Strain ANSB01E. Curr Microbiol [Internet]. 2020;77(2):273–8. Available from: https://doi.org/10.1007/s00284-019-01811-8

35. Li Y, Li X, Jia D, Liu J, Wang J, Liu A, et al. Complete genome sequence and antimicrobial activity of Bacillus velezensis JT3-1, a microbial germicide isolated from yak feces. 3 Biotech [Internet]. 2020;10(5):1–10. Available from: https://doi.org/10.1007/s13205-020-02235-z

36. Deng Q, Wang R, Sun D, Sun L, Wang Y, Pu Y, et al. Complete Genome of Bacillus velezensis CMT-6 and Comparative Genome Analysis Reveals Lipopeptide Diversity. Biochem Genet [Internet]. 2020;58(1):1–15. Available from: https://doi.org/10.1007/s10528-019-09927-z

37. Wu L, Li X, Ma L, Blom J, Wu H, Gu Q, et al. The “pseudo-pathogenic” effect of plant growth-promoting Bacilli on starchy plant storage organs is due to their α-amylase activity which is stimulating endogenous opportunistic pathogens. Appl Microbiol Biotechnol. 2020;104(6):2701–14.

38. Wang B, Liu H, Ma H, Wang C, Kai L, Li Y, et al. Complete Genome Sequence of Biocontroller Bacillus velezensis Strain JTYP2, Isolated from Leaves of Echeveria laui. 2017;5(24):4–5.

39. Reva ON, Dixelius C, Meijer J, Priest FG. Taxonomic characterization and plant colonizing abilities of some bacteria related to Bacillus amyloliquefaciens and Bacillus subtilis. FEMS Microbiol Ecol. 2004;48(2):249–59.

40. Gu G, Gonzalez-Escalona N, Bolten S, Luo Y, Mfaiz AI, Leon MS, et al. Genome Sequences of Brevundimonas naejangsanensis Strain FS1091 and Bacillus amyloliquefaciens Strain FS1092, Isolated from a Fresh-Cut-Produce-Processing Plant. 2020;(November 2019):1–3.

41. Lu JY, Zhou K, Huang WT, Zhou P, Yang S, Zhao X, et al. A comprehensive genomic and growth proteomic analysis of antitumor lipopeptide bacillomycin Lb biosynthesis in Bacillus amyloliquefaciens X030. Appl Microbiol Biotechnol. 2019;103(18):7647–62.

42. Yan Y, Liu S, Wang D, Xue J, Guo D, Song X, et al. Complete genome sequence of Bacillus amyloliquefaciens B15 isolated from grape skin, a strain of strong inhibitory activity against fungi. J Biotechnol [Internet]. 2016;228:28–9. Available from: http://dx.doi.org/10.1016/j.jbiotec.2016.04.036

43. Tran TD, Huynh S, Parker CT, Hnasko R, Gorski L, McGarvey JA. Complete Genome Sequences of Three Bacillus amyloliquefaciens Strains That Inhibit the Growth of Listeria monocytogenes In Vitro. Genome Announc. 2018;6(1):1–2.

44. He P, Hao K, Blom J, Rückert C, Vater J, Mao Z, et al. Genome sequence of the plant growth promoting strain Bacillus amyloliquefaciens subsp. plantarum B9601-Y2 and expression of mersacidin and other secondary metabolites. J Biotechnol [Internet]. 2012;164(2):281–91. Available from: http://dx.doi.org/10.1016/j.jbiotec.2012.12.014

45. Xie S, Vallet M, Sun C, Kunert M, David A, Zhang X, et al. Biocontrol Potential of a Novel Endophytic Bacterium From Mulberry (Morus) Tree. Front Bioeng Biotechnol. 2020;7(January):1–13.

46. Magno-Perez-Bryan MC, Martinez-Garcia PM, Hierrezuelo J, Rodriguez-Palenzuela P, Arrebola E, Ramos C, et al. Comparative genomics within the bacillus genus reveal the singularities of two robust bacillus amyloliquefaciens biocontrol strains. Mol Plant-Microbe Interact. 2015;28(10):1102–16.

47. Borriss R, Chen XH, Rueckert C, Blom J, Becker A, Baumgarth B, et al. Relationship of Bacillus amyloliquefaciens clades associated with strains DSM 7 T and FZB42 T: A proposal for Bacillus amyloliquefaciens subsp.

amyloliquefaciens subsp. nov. and bacillus amyloliquefaciens subsp. plantarum subsp. nov. based on complete gen. Int J Syst Evol Microbiol. 2011;61(8):1786–801.

48. Zhi Y, Wu Q, Xu Y. Genome and transcriptome analysis of surfactin biosynthesis in Bacillus amyloliquefaciens MT45. Sci Rep [Internet]. 2017;7(December 2016):1–13. Available from: http://dx.doi.org/10.1038/srep40976

49. MENG D, ZHAI L xin, TIAN Q peng, GUAN Z bing, CAI Y jie, LIAO X ru. Complete genome sequence of Bacillus amyloliquefaciens YP6, a plant growth rhizobacterium efficiently degrading a wide range of organophosphorus pesticides. J Integr Agric [Internet]. 2019;18(11):2668–72. Available from: http://dx.doi.org/10.1016/S2095-3119(19)62658-4

50. Geng W, Cao M, Song C, Xie H, Liu L, Yang C, et al. Complete genome sequence of Bacillus amyloliquefaciens LL3, which exhibits glutamic acid-independent production of poly-γ-glutamic acid. J Bacteriol. 2011;193(13):3393–4.

51. Zhang G, Deng A, Xu Q, Liang Y, Chen N, Wen T. Complete genome sequence of Bacillus amyloliquefaciens TA208, a strain for industrial production of guanosine and ribavirin. J Bacteriol. 2011;193(12):3142–3.

52. Jeong H, Jeong DE, Kim SH, Song GC, Park SY, Ryu CM, et al. Draft genome sequence of the plant growth-promoting bacterium Bacillus siamensis KCTC 13613T. J Bacteriol. 2012;194(15):4148–9.

53. Kalinowski J, Ahrens B, Al-Dilaimi A, Winkler A, Wibberg D, Schleenbecker U, et al. Isolation and whole genome analysis of endospore-forming bacteria from heroin. Forensic Sci Int Genet [Internet]. 2018;32(October 2017):1–6. Available from: https://doi.org/10.1016/j.fsigen.2017.10.001

54. Heo SJ, Kim JH, Kwak MS, Jeong DW, Sung MH. Functional genomic insights into probiotic bacillus siamensis strain b28 from traditional korean fermented kimchi. Foods. 2021;10(8).

55. Montor-Antonio JJ, Sachman-Ruiz B, Lozano L, del Moral S. Draft genome sequence of Bacillus amyloliquefaciens JJC33M, isolated from sugarcane soils in the Papaloapan region, Mexico. Genome Announc. 2015;3(1):1000000.

56. Dunlap CA. Phylogenomic analysis shows that ‘Bacillus vanillea’ is a later heterotypic synonym of Bacillus siamensis. Int J Syst Evol Microbiol. 2015;65(10):3507–10.

57. Kumar S, Stecher G, Li M, Knyaz C, Tamura K. MEGA X: Molecular evolutionary genetics analysis across computing platforms. Mol Biol Evol. 2018;35(6):1547–9.

58. Tamura K, Nei M. Estimation of the number of nucleotide substitutions in the control region of mitochondrial DNA in humans and chimpanzees. Mol Biol Evol. 1993;10(3).
